# A Scoping Review of the Complementary Feeding Practices and Early Eating Experiences of Children With Down Syndrome

**DOI:** 10.1093/jpepsy/jsad060

**Published:** 2023-09-21

**Authors:** Laura Hielscher, Karen Irvine, Amanda K Ludlow, Samantha Rogers, Silvana E Mengoni

**Affiliations:** Department of Psychology, Sport and Geography, University of Hertfordshire, UK; Department of Psychology, Sport and Geography, University of Hertfordshire, UK; Department of Psychology, Sport and Geography, University of Hertfordshire, UK; Department of Psychology, Sport and Geography, University of Hertfordshire, UK; Department of Psychology, Sport and Geography, University of Hertfordshire, UK

**Keywords:** children, complementary feeding, Down syndrome, feeding problems

## Abstract

**Objective:**

Children with Down syndrome may experience more challenges in their early feeding and may be introduced to complementary foods comparatively later than typically developing (TD) children. This scoping review aimed to identify and synthesize the existing literature that describes feeding problems and early eating experiences relating to the period of complementary feeding for children with Down syndrome.

**Methods:**

Scopus, PubMed, Medline, Web of Science, and PsycInfo were searched. Journal articles published between January 1991 and June 2022 that reported on the complementary feeding period with children with Down syndrome were included.

**Results:**

Eighteen journal articles met the inclusion criteria. Children with Down syndrome were introduced to complementary foods later than TD children and progressed to more challenging food textures at a slower rate. Gross and fine motor skill delays and sensory difficulties contributed to secondary feeding problems such as difficulties chewing, biting, and reduced awareness of food on lips and tongue. Parents of children with Down syndrome reported exercising more caution and employing more controlling feeding practices compared to TD and had higher levels of concern regarding their child’s weight.

**Conclusions:**

Guidelines and early feeding support specific to children with Down syndrome should be available before the first complementary foods are introduced and throughout this period. Feeding support should aim to address parental concerns and provide assistance when feeding problems occur, to minimize delays and encourage the optimum development of eating abilities. Future research should address the development of feeding problems during this period and explore possible interventions.

## Introduction

Down syndrome is a condition which most commonly occurs when a person has an extra copy of chromosome 21 and is generally diagnosed at or before birth. Down syndrome occurs in approximately 1.0–1.5 out of every 1000 live births (Morris & Alberman, 2009; Strippoli et al., 2019) and is the most common genetic cause of intellectual disability. Individuals with Down syndrome will have some degree of intellectual disability and may have a range of anatomical, oral-motor, and structural differences (Cooper-Brown et al., 2008; Field et al., 2003). Children with Down syndrome are at a higher risk of celiac disease, type 1 diabetes, obesity, chronic constipation, thyroid dysfunction, and dental problems (Bergholdt et al., 2006; Bermudez et al., 2019; Michael & Marder, 2021; Oliveira et al., 2010; Pavlovic et al., 2017). Therefore, adequate nutrition is an important concern for this group, to promote positive health outcomes.

Children with Down syndrome may experience more challenges regarding milk feeding and eating solid foods than typically developing (TD) children. It is estimated that the frequency of feeding problems in children with Down syndrome is around 50%–80% (Anil et al., 2019) compared to around 25% in TD children (Manikam & Perman, 2000). For some children with Down syndrome, feeding via nasogastric or gastronomy tube may be required as a result of feeding difficulty or medical complexity (Doshi et al., 2022; Lewis and Kritzinger, 2004; Poskanzer et al., 2020). Cañizares-Prado et al. (2022) attainment of early feeding milestones can be delayed in children with Down syndrome (Anil et al., 2019; Nordstrøm et al., 2020) and as required feeding skills become more complex throughout childhood, they become increasingly delayed in comparison to their TD peers (Nordstrøm et al., 2020).

Some characteristics associated with Down syndrome can contribute to difficulties around feeding in early life. Approximately 40%–60% of infants with Down syndrome are born with a congenital cardiac anomaly, which can disrupt early milk feeding, particularly if surgical intervention is required (Marder et al., 2015; Pisacane et al., 2007). Rates of exclusive breastfeeding are lower for infants with Down syndrome than for TD infants (Magenis et al., 2022), and infants with Down syndrome are more frequently bottle fed than TD infants (Pisacane et al., 2007). The presence of a cardiac anomaly may contribute to infants tiring more easily during feeds and being less likely to display feeding cues. Furthermore, some infants and children with Down syndrome may present with hypotonia (low muscle tone) which can lead to difficulties with positioning during milk feeding and sitting upright when introducing solid foods. Additionally, poor lip seal, difficulty sucking, and an inefficient swallow can lead to choking and aspiration (Agostini et al., 2021). Children with Down syndrome may also present with anatomical differences such as a larger tongue and smaller oral cavity which can lead to abnormal tongue movement (e.g., tongue thrust), pocketing of food and food loss during meals (Ooka et al., 2012).

Delayed motor skill development has been commonly reported in children with Down syndrome (Malak et al., 2015). Delayed oral-motor skills can inhibit the effective manipulation of food in the mouth and the development of the chewing patterns required to eat solid foods safely (Nordstrøm et al., 2020; Overland, 2011). Delayed gross and fine motor skills can hinder the development of self-feeding skills and the ability to use utensils (e.g., knife, fork). Self-feeding and use of utensils are skills that have been identified as delayed in some children with Down syndrome (Anil et al., 2019; Frank & Esbensen, 2015). Difficulties with sensory processing such as oral hyposensitivity and hypersensitivity (reduced/increased response to oral sensory input) are also common in children with Down syndrome, leading to feeding problems such as food refusal, refusal to swallow, selectivity by type and texture, being a “picky eater” and overstuffing food in the mouth (Field et al., 2003; Nordstrøm et al., 2020).

Children with Down syndrome are more likely to receive a diagnosis of autism than the general population, with prevalence estimations for dual-diagnoses ranging from 5% to 41% (Spinazzi et al., 2023; Tevis et al., 2023). Increased feeding and sensory problems are commonly reported among children with autism in comparison to children without autism (Leader et al., 2021). Children who have a dual-diagnosis of Down syndrome and autism are more likely to experience constipation, gastroesophageal reflux (GERD) and feeding difficulties than children with a diagnosis of Down syndrome only (Spinazzi et al., 2023).

The World Health Organization (WHO) recommend that infants begin to receive complementary foods at 6 months of age, alongside breast milk. Between the ages of 6 and 24 months, infants transition from an exclusively milk-based diet to one consisting mainly of solid foods (WHO, 2022). Beginning complementary feeding before a child is developmentally ready may increase the risk of choking, picky eating, food selectivity, obesity, and diabetes (Clayton et al., 2013; Johnson et al., 2018). However, if children begin complementary feeding too late, they may be at an increased risk of malnutrition, stunted growth and micronutrient deficiencies (Green Corkins & Teague 2017). It is recommended that parents offer children a range of gradually increasing textures and flavors, introduced at an appropriate rate (NHS, 2022). This aims to advance the child’s tolerance of increasingly difficult food textures and encourage oral-motor skill development (Alcock, 2006; Schwartz et al., 2011). Importantly, a 2009 longitudinal study conducted with TD children in the United Kingdom found that children who were introduced to solid, lumpy textured food late (after 9 months) ate a less varied diet and had more eating problems at 7 years of age than children who began to eat this texture earlier (i.e., between 6 and 9 months of age; Coulthard et al., 2009). The researchers hypothesized that there could be a sensitive period for the introduction of lumpy textured foods between the ages of 6 and 10 months.

Infants with Down syndrome are likely to be introduced to complementary foods later than TD children (Cochran et al., 2022; Hopman et al., 1998). However, no official guidelines exist for the introduction of complementary foods for infants with Down syndrome specifically, nor is there a gold standard of how to address feeding problems should they occur during this important phase in development. Additionally, existing feeding support services do not meet the needs of some mothers of infants with Down syndrome (Cartwright & Boath, 2018; Hielscher et al., 2022) and families of infants with Down syndrome are more likely to report unmet care needs generally (McGrath et al., 2011). While several reviews have been undertaken regarding breastfeeding infants with Down syndrome (e.g., Magenis et al., 2022; Sooben, 2012; Zhen et al., 2021) offering insights into barriers, facilitators and helpful implications for policy and practice, the same is not the case for complementary feeding.

Given the importance of the complementary feeding period for significant eating, developmental and health outcomes, and the increased likelihood of feeding problems and health complications for children with Down syndrome, this is an important research area that needs to be further explored. This scoping review aimed to identify and synthesize existing literature that describes feeding problems and early eating experiences during the period of complementary feeding for children with Down syndrome.

## Methods

An initial literature search informed the research questions and tested search terms for suitability. This identified a lack of studies that have investigated complementary feeding in infants with Down syndrome. It was determined that a scoping review would be most suitable to provide a broad overview of this research area. The protocol for this scoping review was developed using the framework outlined by Arksey and O'Malley (2005), with enhancements from Levac et al. (2010). The protocol was pre-registered on the Open Science Framework (and can be accessed at: https://osf.io/v5q6k). The study was conducted and reported in line with the PRISMA extension for scoping reviews (Tricco et al., 2018). A final version of the PRISMA-ScR reporting checklist can be found within the Supplementary materials.

At the time of pre-registration, it was intended that this review would investigate the first introduction of solid foods to infants with Down syndrome and feeding problems during this time specifically. However, initial searches identified only two studies that have specifically explored this topic, which would have been insufficient to conduct a full review. A decision was made to expand the focus of this review to encompass the complementary feeding period more broadly (the gradual introduction of new textures and flavors, and gradual reduction of milk consumed after solid foods are first offered to the child, typically up to age 2 but acknowledging that this may be longer in children with Down syndrome) and the development of eating behavior during this time (Birch & Doub, 2014).

This scoping review aimed to address four research questions:

1. What is the reported process of introducing complementary foods for children with Down syndrome?
2. What are the reported feeding difficulties that occur during the complementary feeding period in this population?
3. What factors are associated with increased or reduced feeding problems in this population?
4. What are the research and knowledge gaps regarding the complementary feeding period and early eating experiences of children with Down syndrome?

### Search Strategy

Searches were initially conducted in June 2021 and were re-run in June 2022 using Scopus, PubMed, Medline, Web of Science, and PsycInfo (see Table I for search terms). The same search terms were entered into Google Scholar and the first 200 results were reviewed (in line with recommendations by Haddaway et al. (2015) regarding the use of Google Scholar for evidence reviews). Search alerts were set-up using the same search terms and databases, and they were monitored for new publications from June 2022 to February 2023.

**Table I.** Search Terms Used to Conduct Database Searches

Search Term 1	Search Term 2	Search Term 3
*Search operator:*	*AND*	*AND*
Down syndrome	Infant	Feeding problems
Down’s syndrome	Child	Feeding disorders
Trisomy 21	Children	Feeding difficulties
Intellectual disability		Introduction of solid foods
		Introduction of solids
		Complementary feeding
		Child feeding practices
		Weaning
		Eating behavior

*Note*. The full line by line database search strategy used was: TITLE-ABS (“down syndrome” OR “down's syndrome” OR “trisomy 21” OR “intellectual disability”) AND (“infant” OR “child” OR “children”) AND (“feeding problems” OR “feeding disorders” OR “feeding difficulties” OR “introduction of solid foods” OR “introduction of solids” OR “complementary feeding” OR “child feeding practices” OR “weaning” OR “complementary feeding” OR “eating behaviour”).

Furthermore, websites of relevant Down syndrome organizations were searched to identify relevant articles and information. Key journals were identified (by assessing topic areas for relevance to eating behavior and/or development disabilities) and then manually searched. Reference lists of articles obtained using the search terms were manually scanned to identify further relevant articles.

### Study Selection

Search results were imported into Rayyan. Duplicate records were removed using an online de-duplication tool called Systematic Review Accelerator. Article titles and abstracts were then screened according to the following inclusion criteria:

- Studies that have investigated factors relating to, or which refer to, the complementary feeding period and early eating experiences (relevant to solid foods).
- Studies with participants of any age (although the focus is on complementary feeding, this can occur at varied ages), gender, or geographical location, who are reported to have Down syndrome. As many individuals with Down syndrome have comorbid neurodevelopmental or psychiatric disorders, data from individuals was included regardless of the presence of comorbid disorders. Studies may include parent-report on behalf of the individual with Down syndrome.
- Original articles published in English with either quantitative or qualitative study methodology or design, for example, intervention studies, interviews, case studies. This does not include review articles.
- Studies that also included groups with other diagnoses were included in the review providing that the findings related to the participants with Down syndrome were reported separately.

Articles published prior to 1990, not in the English language or not using human participants and review articles were excluded from the review. The cut-off date of 1990 was selected because initial searches identified that research published before this date was limited and outdated regarding weaning practices described.

Relevant studies that utilized a very broad age range (e.g., 2–18 years) were checked to see if they referred to the complementary feeding period, for example reporting the age at first introduction to solid foods. If they did not explicitly refer to this, and the study results were also not reported separately according to age sub-groups, studies were excluded from the review. In some studies, references to complementary feeding were a small part of what was presented. Where this occurred, only the information relevant to the review’s aims were extracted.

Title and abstract screening was conducted by the primary researcher (L. Hielscher) and 10% of titles were screened by an independent reviewer external to the research team (reviewer 2). During the first stage of screening (title and abstract) reviewer 2 screened 190 articles. There were seven (3.7%) articles in which the reviewers initially disagreed upon inclusion/exclusion. To resolve this, full-text articles were retrieved, and the articles were re-assessed by both reviewers based on the full inclusion criteria, after which both reviewers agreed on whether to include or exclude these articles. During the second stage of screening, full-text articles were then retrieved and reviewed to make a final decision of inclusion (again 10% of full-text articles were reviewed by an independent reviewer). Reviewers 1 and 2 initially disagreed upon the inclusion of three articles. The main reasons for disagreement were due to the age of the study participants (and whether the study findings were relevant to complementary feeding) and whether studies with multiple groups of participants should be included if they did not present findings for children with Down syndrome separately to other groups. Discussions were had with the wider team and ultimately one of these articles were then included in the review. Reviewer 1 also conferred with the wider research team over the relevance of three further articles, and ultimately one of those articles went on to be included in the review.

### Data Analysis

A data extraction table was created using MS excel (see Table II for a summary of selected studies). The primary researcher (L. Hielscher) piloted this table using three studies, to identify areas for improvement and validate the table. The extracted data were reviewed and organized according to several themes which were determined using the research questions, and then further developed based upon the findings of the included studies. The themes were age at starting complementary feeding, difficulty of different food textures, factors affecting eating development during the introduction of solids, parental feeding practices, and their impact on eating development. These themes are operationally defined in the data extraction table, which can be found in the Supplementary materials. Results of this analysis are presented in both narrative and table formats (see Table II). To identify research gaps in this area, the included studies were appraised regarding their methodologies, findings, and recommendations for future research made in the discussion section of each paper. This was reviewed to identify patterns and commonalities across the available research on this topic and identify what is missing. This is incorporated into the *Discussion* section of this article.

**Table II.** Summary of Research Relating to Complementary Feeding and Early Eating Experiences in Children With Down Syndrome

Author(s) (Year of Publication)	Participants, Gender, Age, Diagnosis	Purpose	Method	Results
Al-Sarheed (2006)	225 parents of a school-age child with Down syndrome.^a^ Mothers’ mean age 37.92 years, *SD =* 7.89 years, Fathers’ mean age 45.38 years (*SD =* 11.29 years).	Investigate breastfeeding patterns and introduction to solid foods for children with Down syndrome.	Parent-report questionnaire.	Solid foods introduced at mean age of 7.73 months. 16.4% (*n* = 37) of parents introduced solid foods to their child with Down syndrome at <6 months of age. 45.8% (*n* = 103) introduced solid foods between 6 and 9 months. 37.8% (*n* = 85) introduced solid foods between 9 and 12 months.
Anil et al. (2019)	17 children with Down syndrome aged 2–7 years^b^ (7 males, 10 females) and their parents; 47 TD children (27 males, 20 females) and their parents. Groups matched according to age and socioeconomic status.	Assess feeding and swallowing problems of children with Down syndrome. Assess the impact of feeding problems on the physical, functional, and emotional domains in children with Down syndrome.	Parent-report questionnaires (including a newly developed questionnaire to assess feeding problems and two standardized measures; the Com-DEALL checklist to assess oral-motor skills in toddlers and the Feeding Handicap Index for Children), video-recorded mealtime.	Children with Down syndrome observed to: have significantly more feeding problems than TD in all phases of swallow have difficulty transitioning to varied textured food (35.3%) and chewing solid and semi-solid foods (47%). have a developmentally immature chewing pattern (52.9%). have more difficulty manipulating food in the mouth and swallowing. have greater difficulty chewing and biting solid foods than with liquids. have more physical, functional, and emotional difficulties with feeding than TD group.
Barreiro et al. (2022)	68 children with Down syndrome aged 2–7 years (*M* = 4.6 years, *SD* = 1.8 years; 41 males, 27 females) and their parents.	Examine self-reported feeding practices of parents of children with Down syndrome, and compare this to previous research conducted with TD populations. Identify any relationships between parent ethnicity and demographic factors and child Feeding practices. Determine whether feeding practices are correlated with child weight.	Parent-report questionnaire (updated version of Child Feeding Questionnaire and demographics questions).	Parents of children with Down syndrome reported higher perceived responsibility, lower concern about child weight and restriction in comparison to data reported among the literature for TD children.
Cochran et al. (2022)	22 parents of children with Down syndrome aged 1–5 years,^b^ 8 health professionals	Explore caregiver experiences of introducing complementary foods to children with Down syndrome. Described training received by health professionals on introducing complementary foods and advice they give.	Interviews with parents and health professionals.	Parental themes: (1) differences in feeding practices for children with Down syndrome; (2) limited guidance and decisions to not specifically follow recommendations; (3) feeding difficulties and related stress; (4) gross motor milestone acquisition related to feeding milestones. Health professionals’ themes: (1) limited practitioner resources/training; (2) providing similar recommendations as for children without Down syndrome; (3) desire for training/resources.
Collins et al. (2003) ^c^	262 children with Down syndrome aged 2-18 years (*M* = 7.99 years, *SD* = 4.18 years), and their TD siblings (*N *= 167), 107 children with autism (and TD siblings [*n* = 69]), 36 children with Cri Du Chat syndrome [and TD siblings (*n* = 14)]. Participants split into age groups for analysis: 2–4.99 years, 5–9.99 years and 10–19.9 years.	Assess eating behaviors of children with certain diagnoses compared to TD siblings. Described eating behaviors, ability to cope with range of textures in family diet and self-feeding/drinking skills and assess implications for development of oral-motor and communication skills.	Parent-report questionnaire.	Children with Down syndrome aged 2–4.99 years: had poorer self-feeding skills than TD siblings and older children. 15% of this group reported to finger feed or need feeding, and 10% reported to have mastered drinking skills. 15% reported to swallow without chewing (TD siblings less likely to do this). Children with Down syndrome more likely to display problem behavior during mealtimes, for example, eating too slow/fast, playing with food, taking food from others' plates, than TD siblings.
Collins et al. (2004) ^c^	262 children with Down syndrome aged 2-18 years (*M* = 7.99 years, *SD****=*** 4.18 years), and their TD siblings (*N* = 167), 107 children with autism spectrum disorder (and TD siblings [*n* = 69]), 36 children with Cri Du Chat syndrome and TD siblings (*n* = 14). Participants split into age groups for analysis: 2–4.99 years, 5–9.99 years and 10–19.9 years.	Described usual meal, snacking patterns and food choices of sample.	Parent-report questionnaire.	For children with Down syndrome in 2–4.99 years age group: food choice was often of soft, sticky, sweet food and sweet beverages. 48.5% (*n* = 66) of this group ate ice cream every day compared to 26.3% of their TD siblings.
Field et al. (2003)	349 children with and without developmental disabilities aged 1 month- 12 years^a^ who were assessed for feeding problems in clinic (200 male, 149 female). 225 of 349 children were identified as having a developmental disability of which type and severity varied widely. Three sub-groups of developmental disability analyzed separately: Autism (*n* = 26), Down syndrome (*n* = 21), Cerebral palsy (*n* = 44.) Authors report that 81% of sample were aged 5 years or younger. Groups were not age matched.	Identify possible predisposing factors for specific childhood feeding problems.	Review of clinic medical records of children who were assessed for feeding problems over a 30-month period.	Many children with Down syndrome refused to chew despite being able to and so ate low-textured purees. Prevalence of different problems for children with Down syndrome were: • Oral-motor delays—80% • Selectivity by texture—45% • Dysphagia—36% • Food refusal—6% • Selectivity by type—5% Issues with oral-motor delays and selectivity by texture were higher than the other groups.
Hopman et al. (1998)	44 children with Down syndrome aged 0-4 years (22 male, 22 female, *M = *21 months, *SD*= 11 months), 37 TD children (19 male, 18 female, *M = *22 months, *SD=* 13 months)	Investigate nutritional status, breastfeeding patterns, age of introduction to solids, energy and nutrient intakes of children with Down syndrome compared to TD children.	Height/weight measurements, parental interview with nutritionist, dietary history method and analysis.	Delayed introduction of solid foods for children with Down syndrome and mean daily energy intake was 27% below recommended daily allowance, compared to 9% below in TD group. Children with Down syndrome received significantly more energy from carbohydrates than recommended daily allowance.
Mohamed et al. (2013)	108 parents of children with Down syndrome aged 5-12 years ^b^ Mean age for males within the Down syndrome group: *M = *8.2 years, *SD=* 1.7 years, mean age for females within Down syndrome group: *M *=* *7.9 years, *SD*= 1.5 years and 113 TD siblings. Mean ages of males and females across Down syndrome and TD groups not significantly different.	Investigate dietary practice and physical activity among children with Down syndrome.	Parent-report questionnaire and interview.	Children with Down syndrome introduced to solid foods later than TD siblings.
Ooka et al. (2012)	17 Children with Down syndrome (9 male, 8 female, *M = *2 years 9 months, *SD=* 9 months). 16 children with autism, 20 children with intellectual disability (age range across all groups was 2 years 2 months–5 years 2 months). Groups did not differ significantly regarding mean age or gender.	Analyze feeding problems reported by caregivers. Evaluate child feeding function.	Review of notes from feeding consultation.	Children with Down syndrome had difficulties with food capturing, chewing, and self-feeding. Frequency of self-feeding lower for children with Down syndrome than other groups. Tongue thrust only seen in children with Down syndrome. Chewing and inappropriate ‘form of meal’ (relating to food texture, e.g., pureed, mashed, soft) most frequently reported feeding problems in Down syndrome group.
Osaili et al. (2019)	83 individuals with Down syndrome^b^ aged 2–19 years (55 males, 28 females) and their parents. Median age of 9 years, interquartile range 8 years. Participants split into age groups for some analyses: 2–4.99 years (*n *= 13), 5-8.99 years, 9–11.99 years, 12–19.99 years.	Assess the physical status, feeding problems, parent-child feeding relationship and weight outcome in children and adolescents with Down syndrome in the UAE.	Questionnaires (standardized measures include STEP-CHILD screening tool for feeding problems and Child Feeding Questionnaire) and anthropometric measurements.	More children with Down syndrome aged 2–4.99 years reported to be dependent on caregivers when eating (84.6%) and to push food away or leave food (53.8%) than all other age groups. Total scores of STEP-CHILD screening tool for feeding problems highest in 2–4.99 years age group. Chewing problems significantly associated with age and decreased as age increased.
Roccatello et al. (2021)	Parents of 34 children with Down syndrome^b^ (median age 7 years, 12 female, 22 male) aged 1–16 years. Children grouped into three age groups: 1–6 years (*n* = 13), 7–12 years (*n* = 16), 13–16 years (*n* = 5).	Investigate eating and lifestyle habits of children with Down syndrome.	Parent-report questionnaire (which included recall of foods eaten across 3-day period), interviews with a dietician.	73% of overall sample first introduced to complementary foods at mean age of 7.5 months (*SD* = 2 months). 27% of children experienced further delays in their introduction to complementary foods (age not reported) and this was attributed to surgery in early life. Issues with texture were also reported by this sub-group, for example, only eating purees, specific difficulties with meat, raw vegetables or fruit. Texture and sensory issues reported across the sample, for example, with solid, hard consistencies (52%), dual-textured meals (48%), fibrous/stick/smelly foods (45%). Reported causes of delayed introduction of complementary foods across the sample were: lack of appetite and unwillingness to chew (50%), food refusal due to taste aversion (18%), finding foods hard to chew (18%). Large variation in age at which sippy cups/drinking glasses introduced (*M = *23 months, *SD = *16 months) and only 21% (*n* = 7) able to independently drink by recommended age (24 months). 53% of parents received specific nutrition counseling after initiating introduction of complementary foods and the remaining 47% received support from elsewhere, for example, internet, peers, health professionals 85% of children completed entire portion sizes offered to them.
Rogers et al. (2022)	40 parents of children with Down syndrome aged 6 months–5 years (*M *=* *30.3 months, *SD* = 15.7 months, 18 female, 22 male) and 40 parents of TD children (*M = *30.5 months, *SD* = 16.0 months, 18 female, 22 male) Groups pairwise matched for age and gender.	Explore feeding problems in young children with Down syndrome, related eating behaviors and parental feeding practices, compared to TD children.	Parent-report questionnaire (standardized measures include Baby Eating Behavior Questionnaire, Child Eating Behavior Questionnaire, Montreal Children’s Hospital Feeding Scale, Child Feeding Practices Questionnaire).	Children with Down syndrome had more feeding problems and solid foods were introduced later (*M* = 6.2 months, *SD* = 1.71 months). Feeding problems negatively associated with general appetite and breast milk duration but positively associated with slowness in eating during exclusive milk feeding. Correlation between feeding problems and food avoidant behaviors in both Down syndrome and TD group. No relationship between feeding problems and parental feeding practices. Feeding problems not related to age of introduction to complementary foods.
Ross et al. (2019)	157 parents of children with Down syndrome aged 11–58 months (*M = *31.5 months, SD not stated, 98 male, 59 female).	Investigate which textures were reported to be easy for the children to eat and which textures were reported to be difficult to eat.	Parent-report questionnaire.	As children got older, dry and hard textures more likely to be reported as easy. As they got older, lumpy, gooey, mushy, wet textures were less likely to be described as easy. Chewy and firm more often reported as difficult.
Ross et al. (2022)	111 parents and children with Down syndrome aged 11-58 months. Children characterized as texture sensitive (TS) or non-texture sensitive (NTS). Mean age of TS children with Down syndrome was 33.9 months, *SD =* 13.1 months, mean age of NTS children with DS was 29.4 months, *SD =* 13.1 months. 107 parents and TD children. Mean age of TD TS group was 28.9 months (*SD =* 8 months), mean age of NTS group was 28.2 months (*SD = *10.5).	Understand mealtime behaviors and identify preferred food textures of children with Down syndrome, using commercial food products.	Video-recorded mealtimes.	Children with Down syndrome less likely to interact with and touch the food overall, compared to TD group. Children with Down syndrome ate less of the food samples overall and were more likely to mouth/suck on food compared to TD group. TS children with Down syndrome showed low disposition toward foods that had loose particles, were grainy, dense, or hard; preferring products that were crispy and dissolvable. Children with Down syndrome who were not texture sensitive liked crisp and dissolvable products with an oily mouthcoating and that were salty and cheesy.
Shaw et al. (2003)	One child with Down syndrome aged 6 years (male).	Described multidisciplinary treatment approach to complex feeding disorder.	Case study/clinical observations.	Solid food introduced after intervention (aged 6 years), variety of foods, tastes and textures accepted, able to eat without distractions and complete meals within reduced time frame (30-min intervals), able to self-feed at school, speech and language improved, weight increased in line with growth charts at 6 months after treatment.
Spender et al. (1996)	Parents of 14 children with Down syndrome aged 11–34 months^b^ (8 female, 6 male). The children with Down syndrome were matched to data from TD children in a previous study on developmental age (assessed using the Bayley Scales of Infant Development).	Investigate various elements of feeding and related factors in infants with Down syndrome in comparison to a TD group.	Video-recorded mealtimes during home visits, including research offering food. Assessed using Feeding Interaction Scale. the Schedule for Oral Motor Assessment. Parent interview. Measurement of weight and height questionnaires (Bates 13-month questionnaire for child temperament; General Health Questionnaire for parental mental health).	Higher proportion of oral-motor dysfunction observed for children with Down syndrome than TD group. Children with Down syndrome had greater challenges regarding oral-motor control and co-ordination of chewing and biting movements. Children with Down syndrome commonly retained food in the mouth without swallowing, showed food loss and were less likely to self-feed than TD comparison group. Seven (50%) mothers described their child with Down syndrome as a fussy eater. TD comparison group more accepting of different types of food. Mealtimes did not significantly differ in length between groups. More parents of children with Down syndrome had sought advice regarding feeding problems Seven (50%) mothers of children with Down syndrome reported difficulties introducing complementary foods. Parents of children with Down syndrome more likely to display non-verbal controlling feeding practices.
van Dijk and Lipke-Steenbeek (2018)	32 parents of children with Down syndrome aged 1–3 years (*M = *21.53 months, *SD=* 7.08 months, 23 males, 9 females).	Compare feeding problems reported by caregivers, in comparison to research conducted with TD children Compare reported feeding problems with observed feeding skills.	Interview, questionnaire (SEP- the Dutch version of the MCHFS, to measure feeding problems), video-recorded mealtime.	Parents of Down syndrome group did not report higher feeding problems than TD norms. Children with Down syndrome displayed little self-feeding and high levels of tongue protrusion while chewing during the recorded mealtime. However, this was not correlated with parental report of feeding problems in the questionnaire.

a Age range, mean, and standard deviation for children with Down syndrome not reported.

b Mean age for children with Down syndrome not reported.

c Collins et al. (2003, 2004) report data and findings collected from the same sample, at the same time.

*Note*. *TD* = typically developing; *TS* = texture sensitive.

## Results

### Included Studies

**Figure 1.**
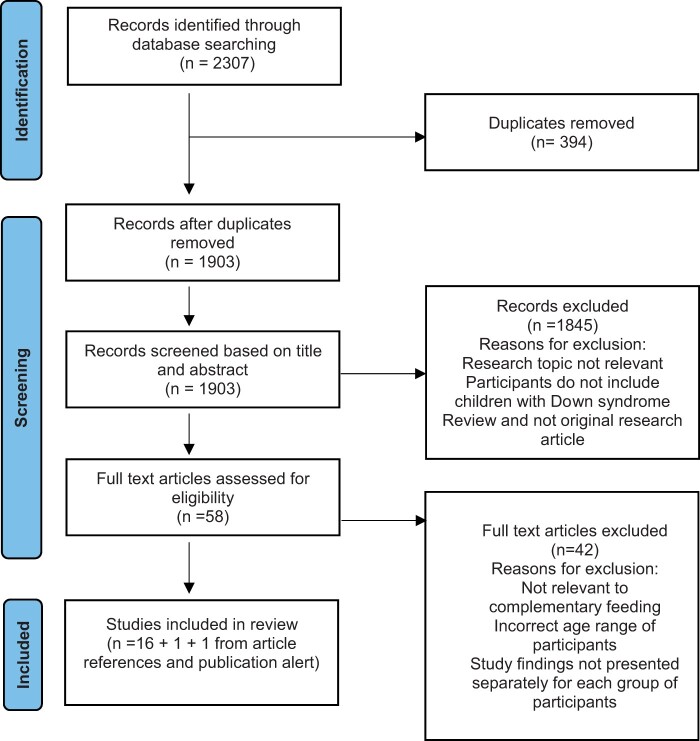
Overview of the study selection process.

Figure 1 shows an overview of the study selection process. Initial searching of electronic databases produced 2307 records. Duplicates were then removed, and title and abstract screening was carried out for 1903 records. Following this, 58 full-text articles were retrieved and assessed for inclusion eligibility, whereby 16 records were identified for inclusion in the review. The references of the 16 records were screened and one article (van Dijk & Lipke-Steenbeek, 2018) was subsequently retrieved for screening and included in the review. Another record was identified via an automated publication alert (Ross et al., 2022), which occurred shortly after the database searches were conducted (June 2022) and was screened and included in the review. In total, 18 records were included in the review.

### Study Characteristics

Table II presents the characteristics of the 18 studies included in the review. Study designs included parent-report questionnaires/surveys, of which six studies used these only (Al-Sarheed, 2006; Barreiro et al., 2022; Collins et al., 2003, 2004; Rogers et al., 2022; Ross et al., 2019). Four studies utilized interviews in addition to conducting parent-report questionnaires (Anil et al., 2019; Mohamed et al., 2013; Osaili et al., 2019; Roccatello et al., 2021). Two studies reported anthropometric measurements (Osaili et al., 2019; Hopman et al., 1998). Mealtimes were video-recorded in four studies (Anil et al., 2019; Ross et al., 2022; Spender et al., 1996; van Dijk & Lipke-Steenbeek, 2018). Other methodologies include a case study (Shaw et al., 2003), and a review of records for children who had previously been assessed for feeding (Field et al., 2003; Ooka et al., 2012).

Six of the studies were conducted in the United States (Barreiro et al., 2022; Cochran et al., 2022; Field et al., 2003; Ross et al., 2019, 2022; Shaw et al., 2003). Two of the studies were conducted in the Netherlands (Hopman et al., 1998; van Dijk & Lipke-Steenbeek, 2018). Four of the studies were carried out in the United Kingdom (Collins et al., 2003, 2004; Rogers et al., 2022; Spender et al., 1996). Two of the studies were conducted in Saudi Arabia (Al-Sarheed, 2006; Mohamed et al., 2013). Other countries that the studies in this review originate from were India (Anil et al., 2019), Italy (Roccatello et al., 2021), Japan (Ooka et al., 2012), and the UAE (Osaili et al., 2019).

### Participants

All 18 studies included children with a diagnosis of Down syndrome and/or their parents. In total, 1276 children with Down syndrome were represented across all of the included studies. Collins et al. (2003, 2004) report data and findings collected from the same sample, at the same time. In eight of the studies, only the parents were the research participants (Al-Sarheed, 2006; Barreiro et al., 2022; Collins et al., 2003, 2004; Mohamed et al., 2013; Roccatello et al., 2021; Rogers et al., 2022; Ross et al., 2019). In one study, participants included parents of children with Down syndrome and healthcare professionals (Cochran et al., 2022). In seven studies, both the parents and the children with Down syndrome were the research participants (Anil et al., 2019; Hopman et al., 1998; Ooka et al., 2012; Osaili et al., 2019; Ross et al., 2022; Spender et al., 1996; Van Dijk & Lipke-Steenbeek, 2018).

In two of the included studies, TD siblings of children with Down syndrome were included (Collins et al., 2003; Mohamed et al., 2013) and three of the studies included children with other developmental disabilities (and/or their parents) including autism, Cerebral Palsy, and Cri Du Chat syndrome (Ooka et al., 2012, Collins et al., 2003; Field et al., 2003). Three of the studies used data collected in previous studies to provide a TD comparison group (Barreiro et al., 2022; Spender et al., 1996; van Dijk & Lipke-Steenbeek, 2018).

The ages of children represented ranged from 0 to 19 years across the studies, but the majority of studies included participants in early childhood. Of the studies that report an age range for participants, eight included children with Down syndrome aged 5 years and under (Cochran et al., 2022; Hopman et al., 1998; Ooka et al., 2012; Rogers et al., 2022; Ross et al., 2019, 2022; Spender et al., 1996; Van Dijk & Lipke-Steenbeek, 2018), with nine studies also including older children. Al-Sarheed (2006) described their sample as school children but do not provide an age range.

Five studies reported medical comorbidities among the participants with Down syndrome. Cochran et al. (2022) note that the parents of 22 children with Down syndrome aged 14–59 months who took part in their study reported that 18 (82%) of the children with Down syndrome had at least one medical comorbidity. Two of the children (9%) had gastronomy feeding tubes in place, and 16 (72%) of the children had previously undergone at least one surgical procedure requiring sedation prior to participating in the study. Ten children had dysphagia (45%), nine (41%) children had cardiac anomalies, and four (18%) children had obstructive sleep apnea. Field et al. (2003) reported that of the 21 children with Down syndrome, 14 (66.7%) had GERD and 14 (66.7%) had cardiopulmonary disease. In the case study conducted by Shaw et al. (2003), the child previously experienced difficulties with lung infections, choking, vomiting, and diarrhea. Little detail is provided about how data on medical comorbidities was collected in these studies.

## What Is the Complementary Feeding Period Like for Children With Down Syndrome? 

### Age at Starting Complementary Feeding

All seven of the studies that measured the age of introduction of complementary foods to infants with Down syndrome reported that for the majority of children this was happening later than six months of age. Mohamed et al. (2013) found the largest proportion of their sample of children with Down syndrome (42.7%) were introduced to complementary foods at 7 months of age or later whereas their TD siblings were most commonly introduced to complementary foods at 4 months of age (53.3%). Al-Sarheed (2006) reported the mean age of introduction to complementary foods was 7.73 months in their sample and 37.8% of the sample began complementary feeding after 9 months. Roccatello et al. (2021) reported that 73% of their sample were introduced to complementary foods at 7.5 months or later, with surgery in early life being a common reason for delaying beyond this age. Furthermore, Rogers et al. (2022) reported that children with Down syndrome were introduced to solid foods significantly later than age-matched TD children at a mean age of 6.2 months compared to 5.49 months. Cochran et al. (2022) also reported the introduction of complementary foods occurring later for children with Down syndrome (6.2 months) compared to their TD siblings (5.1 months) but did not state whether this difference is statistically significant. Of the parents who had multiple children, 60% described introducing complementary foods at an older age than for their TD siblings and parents recalled introducing complementary foods “differently” for their children with Down syndrome, for example, adopting a more cautious approach than they had previously employed with their TD child.

Hopman et al. (1998) found that infants with Down syndrome were introduced to a variety of solid foods later than TD children. For example, the infants with Down syndrome were first introduced to bread at 12 months, compared to 8 months for the TD group. Hard pieces of fruit were introduced at 30 months of age, compared to 12 months for the TD group. The first mixed meal that required chewing (described by the authors as containing vegetables and/or meat and/or starch) was introduced to infants with Down syndrome at 24 months, compared to 12 months for the TD group, indicating that the process of complementary feeding may be longer for children with Down syndrome.

One case study (Shaw et al., 2003) described a child with Down syndrome who was introduced to solid foods as late as 6 years of age, following extensive multidisciplinary treatment to address his aversion to solid foods. Before treatment, the child was exclusively fed PediaSure (a nutritional supplement drink) via a bottle. He very occasionally sampled some solid foods (e.g., ice cream or licking potato chips), but otherwise ate no solid food. His aversion had been exacerbated by previous traumatic medical experiences during attempts to introduce solid foods, such as choking, dehydration, lung infections, and multiple blood tests.

### Difficulty of Different Food Textures

Nine papers commented on the impact of different food textures when starting to eat solid foods, with some textures found to be particularly challenging, and some textures significantly easier, which affected the overall diet of the children.

Spender et al. (1996) reported that children with Down syndrome aged 11–34 months had greater difficulties with all solid textures measured (puree, semi-solid, solid, and crackers) than a TD comparison group; however, this difference was not statistically significant. Roccatello et al. (2021) described four children in their sample who ate only puree textures until 3–4 years of age and noted that meat, raw vegetables, fruit, and dual-textured meals were commonly reported as difficult to eat. Hopman et al. (1998) also found that young children with Down syndrome consume more foods that require less chewing, and eat foods requiring higher chewing skills such as meat, less often than TD children. Some of the parents interviewed by Cochran et al. (2022) described the difficulties that their children with Down syndrome experienced with food textures, as demonstrated by Kristina: “He got really constipated at the beginning. And then that whole spoon in his mouth, he didn’t like that… Dry textures is a huge aversion and anything too chunky. He does gag sometimes if there’s too many pieces, or too chunky, or too thick.”

Ross et al. (2019) found that easier textures included creamy, crispy, crunchy, puree, and soft foods, whereas more difficult textures included chewy and firm. The sample of children ranged from 11 to 58 months and it was observed that as children grew older, some textures became less likely to be reported as easy to eat (such as wet, lumpy, mushy) and some textures such as dry and hard would become easier to eat. In a later study, Ross et al. (2022) used an assessment tool to characterize children with Down syndrome as either texture sensitive (TS, *n* = 91) or non-texture sensitive (NTS, *n* = 127). The assessment tool included statements such as “my child prefers one texture of food” and “my child would rather drink than eat,” and parents rated the frequency of which that statement was true for their child. TS children preferred foods with crispy or dissolvable textures and disliked foods which were grainy, dense or had loose particles. NTS children were found to tolerate a larger number of textures overall than TS children.

The extent of problems related to textures varied across the studies. Field et al. (2003) reported that 45% of the children with Down syndrome showed food selectivity regarding different textures, whereas Anil et al. (2019) reported that 35.3% of children with Down syndrome demonstrated difficulty in transitioning to varied textured food.

In contrast, Collins et al. (2003, 2004) described the majority of the children with Down syndrome aged 2–4.99 years were mostly able to cope with the usual family diet (and therefore did not need foods to be mashed or pureed) and there was a small group of parents in Cochran et al. (2022) who reported that their children were eating very well.

## Contributing Factors Associated With Increased or Reduced Feeding Problems during Complementary Feeding

Twelve studies described feeding problems in infants with Down syndrome. In Cochran et al. (2022), 45% of parents reported feeding difficulties when complementary foods were introduced, and Spender et al. (1996) reported that 63.6% of parents of children with Down syndrome described difficulties when introducing complementary foods. Feeding problems were defined and measured differently across studies, and some feeding problems appear to be interrelated.

### Oral-Motor Skills

Hopman et al. (1998) stated that delayed oral-motor skills can be both a cause and consequence of delayed introduction to solid foods. Roccatello et al. (2021) noted that a “lazy” chewing pattern led to delays regarding the introduction of complementary foods in 45% of children with Down syndrome. Oral-motor delays were linked to food loss, holding food in the mouth longer without chewing, swallowing before food has been chewed sufficiently, incomplete swallowing, difficulties taking an active bite, texture selectivity, and choking or vomiting (Anil et al., 2019; Ooka et al., 2012; Shaw et al., 2003; Spender et al., 1996). Furthermore, in the case study conducted by Shaw et al. (2003), the authors described how the child’s ability to tolerate increasingly difficult and varied food textures was linked to development of his oral-motor skills.

However, when infants with Down syndrome are given simpler textures, for example, soft, pureed foods over a prolonged period of time, their oral-motor development is hindered. Field et al. (2003) report that 82% of their sample of children with Down syndrome had oral-motor delays, and also that a large proportion of the sample ate only pureed or low-textured foods. In some cases, children would refuse to chew despite having the ability to do so, and so their diets consisted mainly of foods that did not require chewing. In their discussion, the authors hypothesized that these children may have developed an aversion to chewing because they associate it with earlier experiences of gagging, vomiting, or choking.

### Gross and Fine Motor Skills

Gross and fine motor skills were reported to affect various elements of self-feeding, for example, eating with fingers or spoon, drinking using straws, not spilling food during meals (Anil et al., 2019; Collins et al., 2003; Shaw et al., 2003; Spender et al., 1996). van Dijk and Lipke-Steenbeek (2018) reported that 31.3% of children with Down syndrome did not self-feed at all, but do not comment on why this may be. Osaili et al. (2019) reported that 84.6% of children with Down syndrome aged 2–4.99 years were unable to eat independently. Children with Down syndrome were found to have increased difficulties using utensils (compared to TD controls) and required specific utensils and positions while feeding and eating (Anil et al., 2019). During parent interviews, Cochran et al. (2022) identified a theme that highlighted relationships between acquisition of gross motor skills and feeding milestones; one mother described her son beginning to crawl at 10 or 11 months old, stating it was also “about the time he started getting better at eating.” When reflecting on their results in their discussion, Collins et al. (2003) highlighted how a child’s ability to cut food into small mouthfuls using a knife and fork may also indirectly impact chewing ability, explaining that small mouthfuls will be much easier to chew and swallow.

### Sensory Difficulties

Oral hypo- and hypersensitivity were also seen to lead to feeding problems. Anil et al. (2019) observed that children with Down syndrome showed reduced awareness of food on lips and tongue and stuffing of food in the mouth, which was attributed to oral hyposensitivity. They concluded that sensory difficulties hindered eating development by making it more difficult for children with Down syndrome to transition to food of different textures. Additionally, Ross et al. (2022) described that children with Down syndrome were more likely to mouth or suck on food than TD children. They noted several behaviors among their sample that are associated with oral hypersensitivity, for example, being less likely to touch food with their hands, bite into the food, chew/munch on food, or touch food to lips or tongue.

### Parental Feeding Practices and Their Impact on Eating Development

Eight studies described parental feeding practices and how they may differ for children with Down syndrome during the period of introducing solid foods. Cochran et al. (2022) found that some parents exercised more caution when introducing complementary foods to their child with Down syndrome in comparison to how they had approached introducing complementary foods to their TD children. One participant (Donna) explained that she became fearful of introducing complementary foods following feeding problems in the child’s early life. As a result, she introduced complementary foods to her child with Down syndrome 9 months later than she did with her TD children. Similarly, when discussing their findings Collins et al. (2003) and Shaw et al. (2003) reflected that parental anxiety (e.g., fear of choking, weight loss or dehydration) may have led to parents restricting the types of food they offer their child, or prevented them from addressing existing feeding routines which are problematic, including an overreliance on foods of age inappropriate textures or negative behavioral responses to solid foods.

Barreiro et al. (2022) stated that parents of children with Down syndrome reported higher perceived responsibility regarding their child’s weight but lower concern about child weight in comparison to a previously studied control group. The authors also found a significant positive correlation between perceived child weight and concern for child weight. Additionally, in Rogers et al. (2022), parents of children with Down syndrome scored significantly higher than parents of TD children for monitoring feeding behaviors, and lower for involvement, emotional regulation, and teaching about nutrition. However, parental feeding practices were not significantly correlated with children’s feeding problems. Spender et al. (1996) found that parents of children with Down syndrome demonstrated more controlling non-verbal behaviors during mealtimes than a TD comparison group.

## Discussion

This scoping review aimed to identify and synthesize existing literature, which describes feeding problems and early eating experiences during the period of complementary feeding for children with Down syndrome. Eighteen studies met the inclusion criteria and were included in the review. The results of this review suggest that the complementary feeding period is different and longer for children with Down syndrome than TD children.

Children with Down syndrome were introduced to solid foods later than TD children and WHO recommendations. Several factors may influence this, including delayed oral-motor development and chewing abilities, parental anxiety (e.g., regarding risk of choking or weight loss) and surgical or medical intervention in early life which can disrupt eating development and lead to food aversions (Cochran et al., 2022; Hopman et al., 1998; Roccatello et al., 2021; Rogers et al., 2022). Once complementary feeding has begun, children with Down syndrome may progress to more challenging textures at a slower rate compared to TD children and development of self-feeding skills may be delayed. These findings are consistent with a recent review by Nordstrøm et al. (2020), which found that as required feeding and eating skills become more advanced, children with Down syndrome show increasing delays in feeding abilities and self-feeding skills compared to TD peers.

Synthesis of the research included in the present review suggests that children with Down syndrome are more likely to experience feeding problems during the complementary feeding period than TD children. For example; difficulties with chewing and swallowing, difficulty manipulating food while it is in the mouth, food loss, holding food in the mouth without chewing, swallowing before food has been chewed sufficiently, incomplete swallowing, choking, vomiting, picky eating, food aversions, reduced awareness of food on lips and tongue, and stuffing of food in the mouth. Some feeding problems were seen to be secondary to other factors. Underlying oral-motor delays and texture sensitivity appear to be contributing factors that can influence the presence of feeding problems during complementary feeding. This is especially pertinent in light of recent research conducted by (Cañizares-Prado et al., 2022) which identified difficulties regarding the introduction of new flavors for 60% of their sample of adults with Down syndrome and difficulties introducing new consistencies for more than 75% of the sample. The authors also described poor chewing among the participants, and that these factors led to limitations in their diets. This demonstrates that challenges regarding chewing and accepting a range of different flavors and textures can be long lasting and may not improve over time. This highlights the importance of monitoring and addressing any feeding problems as early as possible to avoid the potential of consolidating problems that emerge in childhood.

Within the studies included in the present review, parental feeding practices appear to differ for children with Down syndrome compared to TD children, with parents of children with Down syndrome reporting employing more restrictive, cautious or controlling feeding practices and more concern about their child becoming overweight (Barreiro et al., 2022; Cochran et al., 2022; Collins et al., 2003; Rogers et al., 2022; Shaw et al., 2003; Spender et al., 1996). Some parental feeding practices may arise as a consequence of feeding problems. Parents may limit the difficulty of textures that they offer their child out of fear or anxiety regarding risk of choking or vomiting. However, limiting the difficulty of the textures offered may inhibit the development of their child’s chewing abilities (Schwartz et al., 2011). This demonstrates the importance and necessity of readily available feeding support for parents throughout the complementary feeding period.

It can be incredibly difficult for parents when their child with Down syndrome is experiencing problems with feeding and swallowing (Cochran et al., 2022; Hielscher et al., 2022). Parental concerns and wellbeing must be taken into account when a care plan is being developed (Arslan, 2023). Families of infants with Down syndrome are already noted to have difficulties accessing feeding and medical support that meets their needs (Hielscher et al., 2022; McGrath et al., 2011). To improve feeding support services moving forwards, early and ongoing guidance and feeding support from health professionals is vital and should be available before the first complementary foods are introduced and throughout this period. This would give parents an opportunity to express concerns, a place to seek reassurance and would facilitate early intervention, encouraging optimum development of eating abilities.

The final aim of the present review was to identify gaps in this research area. Only 18 studies met the inclusion criteria, highlighting the paucity of research which has explored the early eating experiences and complementary feeding period for children with Down syndrome. Only one study (Cochran et al., 2022) included health professionals who support families of children with Down syndrome. There were only two studies which explored interventions for problems during complementary feeding, and outcomes (Cochran et al., 2022; Shaw et al., 2003). There is also no longitudinal research examining early eating experiences and complementary feeding for children with Down syndrome. This is an area where video-recorded mealtimes could be utilized (alongside other quantitative measures) to understand how eating behavior at mealtimes develops over time. Furthermore, while several of the included studies described texture sensitivities and their impact on feeding, there is no research which has explored how texture sensitivities develop over time in Down syndrome, nor how they may be effectively addressed. Although some studies reported that parents may have offered children developmentally “easier” textures (Collins et al., 2003; Shaw et al., 2003), there are little rich data around the concerns of parents, feeding support received throughout the complementary feeding period, and what strategies parents employed in response to feeding difficulties. These gaps represent valuable areas that future research could explore.

Furthermore, the studies included in this review were conducted in a wide range of countries and thus represent various individual cultures. Cultural differences can impact infant and child feeding practices, for example, the choice of first solid foods offered to children, the age at which children are first offered solid foods, weaning practices (such as infant-led or more parent-controlled methods), the offering of utensils and development of self-feeding or hand feeding (Pak-Gorstein et al., 2009). The individual studies included in the present review did not comment on the feeding practices specific to the country of origin and how these may have been reflected in the study findings. Future research could examine the impact of cultural differences in feeding practices upon complementary feeding, generally and for children with Down syndrome specifically.

It must be noted that while the studies included in the review originate from a wide range of countries, the vast majority are western countries. Thirteen of the 18 studies in the present review were conducted in western countries, with 10 studies carried out in the United Kingdom and United States. This could be due to the search strategy being limited to original research articles that have been published in English. This may have influenced the review findings, particularly regarding timing of introduction to solid foods and parental feeding practices. It is evident that more research is necessary from a more diverse range of countries and cultures in order to increase confidence in conclusions drawn about complementary feeding and early eating experiences for children with Down syndrome.

A limitation of this review is that many of the studies include small sample sizes, which can be a common practical difficulty when conducting research with populations with developmental disabilities. Despite this, there is agreement between the studies regarding the key findings of this review. Additionally, 6 of the 18 articles included in the review recruited participants with Down syndrome via feeding clinics or another medical setting relevant to feeding intervention, and this may have influenced the studies’ findings, although the findings of these studies do not vastly contrast the findings of studies whereby participants were recruited via different methods.

Moreover, there is limited demographic information provided within the studies regarding the diversity of research participants—only two studies reported detailed demographic information about the sample (Barreiro et al., 2022; Rogers et al., 2022). It is not possible to identify how representative the participant samples are of the Down syndrome population. Factors such as gender of parent respondents, socioeconomic status, childcare setting, parent education, and employment may influence food availability, parental feeding practices and access to professional feeding support (Boak et al., 2016; Scaglioni et al., 2018). Therefore, future research should report more detailed information on individual parental characteristics and how these may influence both child feeding and parental responses to feeding problems. Specifically, future research could explore the implications of childhood feeding problems within other childcare settings and caregivers (e.g., at nursery, pre-school). It is also important to explore how eating behavior and mealtimes may differ across caregivers and childcare settings for children with Down syndrome, and the role this may play in addressing or maintaining feeding problems (Hughes et al., 2007; Patrick & Nicklas, 2005).

Another limitation of the data included in this review is the limited consideration of participants with dual-diagnoses. For example, dual-diagnoses of Down syndrome and autism are common, and children with a dual-diagnosis may be more likely to experience challenges around feeding than children with a singular diagnosis of Down syndrome (Spinazzi et al., 2023). Only five of the included studies reported comorbid medical conditions of participants, and there are limited data around how study findings may have differed for any children within the sample with specific medical conditions or dual-diagnoses.

A limitation of the review process itself is the difficulty determining a cut-off participant age whereby studies would be describing childhood eating generally, and therefore not the complementary feeding period specifically. The WHO suggest that the complementary feeding period lasts from six months to two years of age in TD children. Studies with relevant methodology that included children older than this were included if they made reference to the complementary feeding period or the introduction of solid foods specifically.

This scoping review was conducted in line with the framework originally described by Arksey & O'Malley (2005), and later revised by Levac et al. (2010) which aims to improve the methodology set out by the former. Scoping reviews are exploratory in nature and aim to map a particular area of literature. They do not undertake a risk of bias assessment for studies included in the review, and therefore do not aim to draw clinical conclusions (Khalil et al., 2021; Munn et al., 2018). The present review has enabled us to map the literature about the introduction of complementary food in children with Down syndrome and potential problems likely to occur, but systematic reviews and/or future research such as randomized controlled trials are needed to draw clinical conclusions.

## Conclusions

Based on the literature included in the present review, children with Down syndrome tend to be introduced to complementary foods later than TD children and WHO recommendations. Once the complementary feeding period begins, progression to more challenging food textures occurs at a slower rate than TD and the development of self-feeding skills may be delayed in comparison. Parents may limit difficulty of textures out of fear and anxiety regarding choking or vomiting. Throughout the complementary feeding period children with Down syndrome are more likely to experience feeding problems than TD children.

### Implications

While the profile of Down syndrome may share some similarities with other developmental disorders, children with Down syndrome often present with oral, anatomical and dental differences that can additionally impact feeding and eating development (Cooper-Brown et al., 2008). As such, guidelines for the introduction of complementary foods that are specific to children with Down syndrome are required (Cochran et al., 2022) with further research needed to develop and evaluate the clinical recommendations included. As a general principle, parents should receive guidance before they start to introduce solid foods and should have ongoing easily accessible support from health professionals during the complementary feeding period. This would allow parents to express concerns and facilitate early intervention if problems occur. This may also help parents who are anxious about introducing complementary foods to their child with Down syndrome to feel more confident and attempt to introduce them earlier. Future research should aim to explore the complementary feeding period for children with Down syndrome in further detail. In particular, greater understanding is needed about how texture sensitivities develop over time, how parents adapt to feeding challenges, their concerns during this time, where they access support and to what extent support received meets their needs. Such literature is more widely available for other developmental disabilities—in particular, guidelines and information regarding the effective assessment and treatment of feeding problems for children with autism (Esposito et al., 2023; Hodges et al., 2023). That literature could guide and inform future research into feeding problems and early eating experiences for children with Down syndrome to begin to address some of the gaps identified in the present review.

## Supplementary Material

jsad060_Supplementary_Data

## Data Availability

Data available on request.
